# *BRCA* and Beyond: Impact on Therapeutic Choices Across Cancer

**DOI:** 10.3390/cancers17010008

**Published:** 2024-12-24

**Authors:** Joshua Zhi Chien Tan, Zewen Zhang, Hui Xuan Goh, Joanne Ngeow

**Affiliations:** 1 Division of Medical Oncology, National Cancer Centre Singapore, 30 Hospital Blvd, Singapore 168583, Singapore; joshua.tan.z.c@singhealth.com.sg (J.Z.C.T.); zhang.zewen@singhealth.com.sg (Z.Z.); 2 Cancer Genetics Service, Division of Medical Oncology, National Cancer Centre Singapore, 30 Hospital Blvd, Singapore 168583, Singapore; 3 Lee Kong Chian School of Medicine, Nanyang Technological University, Singapore, 11 Mandalay Rd, Singapore 308232, Singapore

**Keywords:** *BRCA*, hereditary breast and ovarian cancer, PARP inhibitor, prostate cancer, breast cancer, ovarian cancer, pancreatic cancer, targeted therapy, homologous recombination repair

## Abstract

Background: Identifying patients with g*BRCA*m is crucial to facilitate screening strategies, preventive measures and the usage of targeted therapeutics in their management. This review examines the evidence for the latest predictive and therapeutic approaches in *BRCA*-associated cancers. Clinical Description: Data supports the use of adjuvant olaparib in patients with g*BRCA*m high-risk HER2-negative breast cancer. In advanced g*BRCA*m HER2-negative breast cancer, the PARPis talazoparib and olaparib have demonstrated benefit over standard chemotherapy. In ovarian cancer, olaparib, niraparib or rucaparib can be used as monotherapy in frontline maintenance. Olaparib and bevacizumab as a combination can also be used as frontline maintenance. In the relapsed platinum-sensitive setting, olaparib, niraparib and rucaparib are effective maintenance options in *BRCA*m patients who are PARPi naive. Both olaparib and rucaparib are effective options in *BRCA*m metastatic castrate-resistant prostate cancer (mCRPC). Evidence also exists for the benefit of PARPi combinations in mCRPC. In metastatic pancreatic cancer, olaparib can be used in g*BRCA*m patients who are responding to platinum chemotherapy. However, there may be a development of PARPi resistance. Understanding the pathophysiology that contributes to such resistance may allow the development of novel therapeutics. Combination therapy appears to have promising results in emerging trials. Seeking avenues for subsidised genetic testing can reduce the total costs of cancer management, leading to improve detection rates. Conclusion: Identifying breast, ovarian, pancreatic and prostate cancer patients with g*BRCA*m plays a crucial predictive role in selecting those who will benefit significantly from PARPi therapy. The use of PARPi in g*BRCA*m HBOC-related cancers has resulted in significant survival benefits. Beyond *BRCA*1/2, HRR gene assessment and the consideration of other cancer predisposition syndromes may allow more patients to be eligible for and benefit from targeted therapies.

## Simple Summary

The role of detecting germline *BRCA* mutation (g*BRCA*m) has evolved over the years from its primary use in early detection, risk reduction and cascade testing to becoming an important predictive marker that influences cancer treatment. In the treatment of breast cancer, ovarian cancer, prostate and pancreatic cancer, g*BRCA*m plays a crucial role in predicting the benefit of poly (adenosine diphosphate [ADP]—ribose) polymerase (PARP) inhibitors (PARPis). In this review article, we will examine the evidence for the latest predictive and therapeutic approaches in *BRCA*-associated cancers with a focus on the use of PARPis alone or in combination with other treatments in g*BRCA*m. The mechanisms of PARPi resistance, the cost-effectiveness of PARPis as a therapy as well as predictive markers beyond g*BRCA*m will also be discussed.

## 1. Introduction

The hallmarks of carcinogenesis have evolved since their inception to include a personalised approach within the realm of precision medicine and cancer genetics. This usually involves somatic tumour and germline testing for selected individuals to provide targeted therapies and clinical care to maximise efficacy and minimise toxicities. Advancements are constantly being made in exploring novel pathways for cancer treatment to develop personalised care and improve overall outcomes.

It is crucial to identify individuals who may carry cancer predisposition syndromes such as Hereditary Breast and Ovarian Cancer (HBOC) so they can be appropriately managed. This includes screening strategies, preventive measures and the usage of appropriately indicated therapeutics. The most common modality used in germline genetic testing involves blood sampling, although other methods include a buccal swab or skin biopsy. Somatic testing for HBOC usually involves homologous repair deficiency (HRD) testing on tumour samples, reporting on genomic instability score (GIS) and genomic alterations within the tumour cells.

Germline pathogenic variants (PVs) or likely pathogenic variants (LPVs) of *BRCA1/2* genes, inherited in an autosomal dominant pattern, can contribute to HBOC, resulting in an increased lifetime risk of developing breast, ovarian, prostate, pancreatic cancer and melanoma compared to the general population. *BRCA1/2* genes play a crucial role in the homologous recombination repair (HRR) pathway, which is responsible for repairing DNA double-strand breaks. A mutation in a HRR gene can lead to a deficiency in the pathway and contribute to genomic instability, which is one of the hallmarks of carcinogenesis.

Understanding the pathophysiology of HRD has clinical implications such as the selective use of platinum-based chemotherapy in treating carcinomas due to increased sensitivity to inter-strand cross-link breakage. For specific tumour types, the U.S. Food and Drug Administration (FDA) has approved poly (adenosine diphosphate [ADP]—ribose) polymerase (PARP) inhibitors (PARPis) for patients found to have PVs or LPVs in both germline and somatic genes involved in HRR with *BRCA1/2* being one of the more common genes detected. In *BRCA*-deficient or HRD cells, PARPis induce chromosomal instability by the accumulation of chromosomal breaks and eventual lethality via non-homologous end joining, which is a phenomenon termed “synthetic lethality” [1]. This article will review the latest evidence-based management of breast, ovarian, prostate and pancreatic cancer associated with *BRCA1/2* PVs/LPVs as well as discuss some of the challenges in oncology therapeutics.

## 2. Clinical Description

### 2.1. Early Breast Cancer

In the adjuvant treatment of breast cancer, the classes of systemic therapies have evolved from chemotherapy, hormonal therapy and anti-HER2 targeted therapy to include novel targeted therapies including PARPis. The OLYMPIA phase 3 study randomised patients with high-risk triple-negative breast cancer (TNBC) and hormone-positive breast cancer patients with germline *BRCA1/2* mutation (g*BRCA*m) between adjuvant olaparib and placebo for one year [2]. High-risk hormone-positive, HER2-negative breast cancer was defined by having four or more positive lymph nodes in those with no prior neoadjuvant therapy. For those with prior neoadjuvant treatment, patients had to have residual disease and a clinical and pathological stage plus oestrogen receptor and nuclear grade (CPS + EG) score of 3 or more. Those with TNBC and no prior neoadjuvant therapy had a tumour size of greater than 2 cm or positive lymph nodes while patients with prior neoadjuvant therapy were eligible if they had any residual disease. All patients were required to have at least six cycles of chemotherapy in either the neoadjuvant or adjuvant setting and have completed local treatment. At a median follow-up of 3.5 years, 1 year of adjuvant olaparib compared to placebo demonstrated a 4-year invasive disease-free survival (iDFS) benefit of 82.7% versus 75.4%, hazard ratio (HR) 0.63; 95% confidence interval (CI) 0.50–0.78. The 4-year overall survival (OS) was 89.8% compared to 86.4% with a difference of 3.4% HR 0.68; 95% CI 0.47–0.97; *p* = 0.009 [3].

### 2.2. Advanced Breast Cancer

In the advanced setting, the PARPis olaparib and talazoparib have demonstrated survival benefits compared to standard chemotherapy in patients with g*BRCA*m [4,5].

In the phase 3 OlympiAD study, patients with hormone-positive, HER2-negative or TNBC with a g*BRCA*m were randomised between olaparib or a standard chemotherapy regime. Standard chemotherapy regimens included single-agent capecitabine, vinorelbine or eribulin. Patients in this study were required to have prior taxane and anthracycline either in the (neo)adjuvant or advanced setting and no more than two lines of treatment in the advanced setting. The primary endpoint of this study was progression-free survival (PFS) by blinded independent central review (BICR). Compared to a standard chemotherapy regime, olaparib demonstrated a statistically significant benefit with a median PFS of 7.0 months versus 4.2 months, HR 0.58; 95% CI 0.43–0.80; *p* < 0.001 [4]. Olaparib also demonstrated a higher overall response rate (ORR) of 59.9% versus 28.8% in those receiving standard chemotherapy. While there was a numerical trend towards OS benefit with olaparib (median OS 19.3 months versus 17.1 months), this was not statistically significant (HR 0.90; 95% CI 0.66–1.23; *p* = 0.513) [6]. In a prespecified subgroup analysis of patients receiving first-line olaparib in the advanced setting (which constituted 29% of patients in the study), there was an OS benefit for the use of olaparib compared to standard chemotherapy (median OS 22.6 months versus 14.7 months, HR 0.55; 95% CI 0.33–0.95) [7].

In the phase 3 EMBRACA study, patients with similar inclusion criteria to the OlympiAD trial were recruited and randomised between talazoparib and the physician’s choice of chemotherapy (capecitabine, eribulin, vinorelbine or gemcitabine) [5]. Patients were allowed no more than three prior lines of treatment in the advanced setting. The trial also demonstrated a PFS benefit with talazoparib compared to chemotherapy of the physician’s choice (median PFS 8.6 months versus 5.6 months, HR 0.54; 95% CI 0.41–0.71; *p* < 0.001). The ORR of talazoparib was also higher compared to chemotherapy of the physician’s choice (62.6% versus 27.2%). Similar to the OlympiAD study, there was also no OS benefit for Talazoparib as compared to chemotherapy (median OS 19.3 months versus 19.5 months, HR 0.848; 95% CI 0.67–1.07; *p* = 0.17) [8]. The results of both trials are summarised below in Table 1.

One of the major benefits of PARPis as a treatment in advanced g*BRCA*m breast cancer is their oral formulation and relatively well-tolerated side effect profile. One of the limitations of the OlympiAD and EMBRACA studies was the lack of inclusion of platinum chemotherapy as an option for the physician’s choice of chemotherapy. In the TNT trial, carboplatin demonstrated a high ORR of 68% in patients with g*BRCA*m TNBC being treated in the first-line setting [9]. Hence, where cost and availability are an issue, platinum chemotherapy is an alternative to PARPis in patients with g*BRCA*m advanced breast cancer.

### 2.3. Ovarian Cancer

The standard first-line treatment for a newly diagnosed patient with advanced epithelial ovarian cancer is cytoreductive surgery either preceded or followed by platinum-based chemotherapy. Despite the high response rates of platinum chemotherapy in this setting, up to 70% of patients experience a recurrence at 3 years [10]. Hence, the development of maintenance strategies following the completion of platinum chemotherapy has been of utmost importance in reducing recurrence risk. The use of frontline maintenance PARPis has led to significant PFS benefits in multiple phase 3 studies.

The use of olaparib as frontline maintenance in patients with a g*BRCA*m or somatic *BRCA*m was investigated in the SOLO1 phase 3 study [11]. In this study, eligible patients had a partial response (PR) or complete response (CR) following first-line platinum-based chemotherapy and cytoreductive surgery. Patients were randomised between olaparib for 2 years and placebo. For patients with measurable disease and a PR at 2 years, olaparib could be continued. Compared to placebo, olaparib demonstrated a statistically significant PFS benefit (median PFS 56.0 months versus 13.8 months, HR 0.33; 95% CI 0.25–0.43) [12]. At 7 years, the landmark OS analysis showed a benefit that was clinically but not statistically significant (7-year OS 67.0% versus 46.5%, median OS not reached (NR) versus 75.2 months, HR 0.55; 95% CI 0.40–0.76; *p* = 0.0004, *p* < 0.0001 required to declare statistical significance) [13].

In the phase 3 PRIMA study, eligible patients had high-risk advanced disease defined by patients that required neoadjuvant chemotherapy, had inoperable stage III disease, stage III disease with residual disease post-operatively or stage IV disease [14]. Eligibility was selected based on having a CR or PR post-treatment and normalisation of CA 125 levels. Population characteristics showed that 30% of patients had *BRCA*m and 51% had HRD as defined by Myriad MyChoice assay. Patients were randomised between niraparib maintenance for 3 years and placebo. Compared to placebo, niraparib demonstrated superior median PFS in both the HRD subgroup (median PFS 21.9 months versus 10.4 months, HR 0.43; 95% CI 0.31–0.59; *p* < 0.001) and the intention-to-treat (ITT) population (median PFS 13.8 months versus 8.2 months, HR 0.62; 95% CI 0.50–0.76; *p* < 0.001). The PFS at 5 years continued to favour niraparib in both the overall population (5-year PFS 22% versus 12%, HR 0.66; 95% CI 0.55–0.78) and the HRD population (5-year PFS 35% versus 16%, HR 0.51; 95% CI 0.40–0.66). Final OS results showed no statistically significant difference between niraparib maintenance and placebo. Median OS was 46.6 months versus 48.8 months, HR 1.01; 95% CI 0.84–1.23; *p* = 0.88 in the overall population and 71.9 months versus 69.8 months, HR 0.95; 95% CI 0.70–1.29 in the HRD population [15].

In the phase 3 ATHENA-MONO study, patients with advanced ovarian cancer who had undergone surgical cytoreduction and were responding to first-line platinum chemotherapy were eligible [16]. Similar to the PRIMA study, patients were not required to have a *BRCA*m. Patients were randomised between rucaparib for 2 years and placebo. The primary endpoint was that of investigator-assessed PFS in the HRD population (defined by either *BRCA*m or *BRCA* wild type (WT) with a loss of heterozygosity (LOH) score of 16% or greater as assessed by Foundation One next-generation sequencing (NGS)). Population characteristics showed that 40% of the study population were HRD with around 50% of these HRD patients being *BRCA*m, while the other 50% were HRD with *BRCA* WT and high LOH. This trial also demonstrated a statistically significant benefit for rucaparib compared to placebo in the HRD population (median PFS 28.7 months versus 11.3 months, HR 0.47; 95% CI 0.31–0.72; *p* = 0.0004) and ITT population (median PFS 20.2 months versus 9.2 months, HR 0.52; 95% CI 0.40–0.68; *p* < 0.0001).

The above three trials demonstrated the benefit of single-agent PARPis as frontline maintenance therapy. More recently, the use of PARPis in combination with other agents in a doublet or triplet as maintenance therapy has been investigated.

The PAOLA-1 phase 3 study recruited patients with advanced ovarian cancer who had undergone cytoreductive surgery and were responding to platinum chemotherapy and randomised them between olaparib for 2 years together with bevacizumab maintenance and placebo for 2 years together with bevacizumab maintenance [17]. In this study, 50% of the population were HRD positive as assessed by Myriad MyChoice assay, and approximately 29% of the study population had a *BRCA*m. The primary endpoint was that of investigator-assessed PFS in the ITT population. The combination of olaparib and bevacizumab was superior to placebo and bevacizumab with a median PFS of 22.1 months versus 16.6 months, HR 0.59; 95% CI 0.49–0.72; *p* < 0.001 in the ITT population. In patients with tumours positive for HRD, the median PFS was 37.2 months versus 17.7 months, HR 0.43; 95% CI 0.28–0.66 in favour of the olaparib and bevacizumab arm. At 5 years, PFS benefits were maintained in both the ITT population (5-year PFS 29.3% versus 15.8%, median PFS 22.9 months versus 16.6 months, HR 0.63; 95% CI 0.53–0.74) and the HRD population (5-year PFS 46.1% versus 19.2%, median PFS 46.8 months versus 17.6 months, HR 0.41; 95% CI 0.32–0.54). The 5-year landmark OS analysis showed that the OS did not significantly differ between the two arms in the ITT population (median OS 56.5 months versus 51.6 months, HR 0.92; 95% CI 0.76–1.12; *p* = 0.4118) [18]. There was a suggestion of OS benefit in the subgroup of HRD patients including those with *BRCA*m (5-year OS 65.5% versus 48.4%, median OS 75.2 months versus 57.3 months, HR 0.62; 95% CI 0.45–0.85); however, the study was not powered to demonstrate this.

The DUO-O phase 3 study looked at patients with advanced high-grade epithelial ovarian cancer (HGEOC) planned for cytoreductive surgery that were tumour *BRCA* mutation (t*BRCA*m) negative [19]. Approximately 40% of patients were HRD positive, while 55% were HRD negative and 5% had an unknown status. Patients were randomised between three groups; paclitaxel, carboplatin, and bevacizumab followed by bevacizumab maintenance (Arm 1), paclitaxel, carboplatin, bevacizumab, and durvalumab followed by bevacizumab and durvalumab maintenance (Arm 2) and paclitaxel, carboplatin, bevacizumab, and durvalumab followed by bevacizumab, durvalumab and olaparib maintenance (Arm 3). The primary endpoint of this trial was PFS in arm 3 compared to arm 1 in the non-tumour *BRCA* mutated HRD-positive patients and in the ITT population. In the non-tumour *BRCA* mutated HRD positive population, arm 3 demonstrated a statistically significant PFS benefit compared to arm 1 (median PFS 37.3 months versus 23 months, HR 0.49; 95% CI 0.34–0.69; *p* < 0.0001). This was also seen in the ITT population for arm 3 compared to arm 1 (median PFS 24.2 months vs. 19.3 months, HR 0.63; 95% CI 0.52–0.76; *p* < 0.0001). The major limitation of this study was the lack of an arm with only bevacizumab and olaparib, making it challenging to ascertain how much of the benefit was contributed by durvalumab in arm 3. This limitation is important to consider given the treatment costs and toxicities associated with triplet therapy.

In summary, PARPis maintenance with olaparib should be used in patients with *BRCA*m. In patients with HRD, olaparib with bevacizumab, niraparib or rucaparib should be considered as maintenance therapy. The results of these trials are summarised below in Table 2.

In patients with *BRCA*m platinum-sensitive relapsed ovarian cancer who did not receive frontline PARPi therapy, maintenance in the 2nd line setting is an option. In the SOLO2 phase 3 study, patients with platinum-sensitive relapsed ovarian cancer and *BRCA*m that were responding to platinum-based chemotherapy were randomised between olaparib maintenance until progression or placebo [20]. There was a statistically significant PFS benefit with olaparib maintenance compared to placebo (median PFS 19.1 months versus 5.5 months, HR 0.30; 95% CI 0.22–0.41; *p* < 0.0001). While there was a trend towards OS benefit with olaparib (median OS 51.7 months versus 38.8 months, HR 0.74; 95% CI 0.54–1.00; *p* = 0.054), this was not statistically significant [21]. Notably in this study, the rate of secondary myelodysplastic syndrome (MDS) and acute myeloid leukaemia (AML) was higher in the olaparib arm when compared to the placebo arm (8% versus 4%). Possible reasons for this included the longer duration of PARPi treatment and more heavy chemotherapy pre-treatment.

The phase 3 NOVA study randomised patients with platinum-sensitive recurrent ovarian cancer post-completion of platinum-based chemotherapy to niraparib maintenance until progression of disease or placebo [22]. This study included both g*BRCA*m and non-*BRCA*m patients. In the g*BRCA*m population, there was a statistically significant PFS benefit for niraparib maintenance compared to placebo (median PFS 21.0 months versus 5.5 months, HR 0.27; 95% CI 0.17–0.41). OS was not statistically significantly improved in this population. In the *BRCA* WT population, while there was a PFS benefit for niraparib compared to placebo (median PFS 9.3 months versus 3.9 months, HR 0.45; 95% CI 0.34–0.61; *p* < 0.001), there was a trend towards detrimental OS with niraparib (median OS 31.0 months versus 34.8 months, HR 1.06; 95% CI 0.81–1.37) [23]. This suggestion of poorer OS outcomes in the niraparib arm in non-*BRCA*m patients led to an FDA request to restrict the approval of niraparib maintenance to patients with *BRCA*m ovarian cancer in the platinum-sensitive relapsed setting.

The phase 3 ARIEL3 study randomised platinum-sensitive relapsed ovarian cancer patients to rucaparib maintenance until progression or placebo [24]. This study also showed a statistically significant PFS benefit for patients with *BRCA*m who were randomised to rucaparib as compared to placebo (median PFS 16.6 months versus 5.4 months, HR 0.23; 95% CI 0.16–0.34; *p* < 0.0001). OS was also not statistically significant in this trial population [25]. In the ITT population which included non-*BRCA*m patients, there was a PFS benefit for maintenance rucaparib. However, similar to the ARIEL3 trial, there was a trend towards poorer OS outcomes in the ITT population with rucaparib (median OS 36.0 months versus 43.2 months, HR 0.995). This also led to an FDA request to restrict the approval of 2nd line maintenance rucaparib to *BRCA*m patients only.

In patients with recurrent *BRCA*m platinum-sensitive ovarian cancer without prior PARPi exposure, maintenance therapy with olaparib, niraparib or rucaparib improves PFS and should be discussed. Frontline PARPi maintenance in patients with *BRCA*m is the preferred approach however, given the significant benefits of PARPi maintenance, the limited duration of treatment and the lower rates of MDS/AML when used in this setting.

Studies have also looked at the comparison of PARPi treatment versus chemotherapy in platinum-sensitive relapsed ovarian cancer with g*BRCA*m. The SOLO3 phase 3 trial compared olaparib versus chemotherapy of physician’s choice [26] while the ARIEL4 phase 3 trial compared rucaparib versus chemotherapy in this setting of platinum-sensitive relapsed ovarian cancer with two or more prior lines of chemotherapy treatment [27]. While both trials showed a PFS benefit for the PARP inhibitor as compared to chemotherapy, the subsequent analysis of OS for patients with three or more prior lines of chemotherapy in SOLO3 and those with two or more prior lines of chemotherapy in ARIEL4 showed a detrimental effect on OS with PARP inhibitor treatment. This led to the withdrawal of late-line indications for PARPi treatment. The American Society for Clinical Oncology (ASCO) guidelines have also recommended against using PARPis as monotherapy in platinum-sensitive recurrent epithelial ovarian cancer [28].

### 2.4. Prostate Cancer

In metastatic prostate cancer, both germline and somatic genetic testing are recommended by the ASCO and National Comprehensive Cancer Network (NCCN) guidelines.

In the setting of metastatic castrate-resistant prostate cancer (mCRPC), multiple PARPis have been evaluated as monotherapy or in combination with novel hormonal agents (NHAs). In the phase 3 PROfound study, patients with mCRPC with progression on at least one NHA and up to 1 prior taxane treatment were randomised between olaparib and the physician’s choice of abiraterone or enzalutamide [29]. Patients were required to have either somatic or germline HRR gene mutations (HRRm). Cohort A of this study included patients with *BRCA1/2* or *ATM* mutations. Cohort B included patients with a mutation in one of the other HRR genes (*BARD1*, *BRIP1*, *CDK12*, *CHEK1*, *CHEK2*, *FANCL*, *PALB2*, *PPP2R2A*, *RAD51B*, *RAD51C*, *RAD51D* or *RAD54L*). In the primary endpoint of imaging-based PFS (ibPFS) in cohort A, olaparib was superior to the control arm (median ibPFS 7.4 months versus 3.6 months, HR 0.34; 95% CI 0.25–0.47; *p* < 0.001). There was also a significant benefit for olaparib in the overall population comprising cohorts A and B (median ibPFS 5.8 months versus 3.5 months, HR 0.49; 95% CI 0.38–0.63; *p* < 0.001). At the final analysis, the median OS in cohort A was 19.1 months with olaparib versus 14.7 months with control (HR 0.69; 95% CI 0.50–0.97; *p* = 0.02) [30]. In the overall population, the median OS was 17.3 months with olaparib versus 14.0 months with control, which was not statistically significant (HR 0.79; 95% CI, 0.61–1.03). Crossover was permitted, and 67% of patients in the control arm of Cohort A and 66% of patients in the overall population subsequently received olaparib on progression. When a sensitivity analysis that adjusted for crossover was conducted, the HR for OS in cohort A was 0.42 (95% CI 0.19–0.91) in favour of olaparib.

In the phase 3 TRITON3 study, patients with mCRPC and a germline or somatic *BRCA1/2* or *ATM* mutation were randomised between rucaparib and physician’s choice of therapy (abiraterone, enzalutamide or docetaxel) [31]. The primary endpoint of the trial was ibPFS. The median ibPFS in the *BRCA*m subgroup was 11.2 months in the rucaparib arm versus 6.4 months in the control arm, HR 0.50, 95% CI 0.36–0.69. In the ITT population, the median ibPFS was 10.2 months in the rucaparib arm versus 6.4 months in the control, HR 0.61; 95% CI 0.47–0.80; *p* < 0.001. In an exploratory analysis of the *ATM* subgroup, the median ibPFS was 8.1 months with rucaparib versus 6.8 months in the control group, HR 0.95; 95% CI 0.59–1.52. At 62 months follow-up, an interim analysis of OS in the *BRCA*m subgroup showed a median OS of 24.3 months versus 20.8 months, HR 0.81; 95% CI 0.58–1.12; *p* = 0.21 for rucaparib versus control. In the ITT population, the median OS was 23.6 months versus 20.9 months, HR 0.94; 95% CI 0.72–1.23.

Doublet therapy including a PARPi also has been studied in several phase 3 trials. In the PROpel phase 3 study, patients were randomised between abiraterone and olaparib and abiraterone and placebo in the first-line mCRPC setting [32]. Patients could be enrolled regardless of HRRm status. Patients were permitted to have received prior docetaxel in the metastatic castrate-sensitive prostate cancer (CSPC) setting. No prior NHA use was permitted unless prior NHAs were stopped for more than 12 months before enrolment. The primary endpoint was ibPFS by investigator assessment. Median ibPFS was 24.8 months in the abiraterone and olaparib arm versus 16.6 months in the control arm, (HR 0.66; 95% CI 0.54–0.81; *p* < 0.001). Both the HRRm and HRR gene non-mutated populations had benefits in the subgroup analysis, although the benefit appeared to be greater in the HRRm. In the HRRm population, the median ibPFS assessed by the investigator was NR versus 13.9 months, HR 0.50; 95% CI 0.34–0.73. In the HRR gene non-mutated subgroup, the median ibPFS assessed by the investigator was 24.1 months versus 19.0 months, HR 0.76; 95% CI 0.60–0.97. The OS at the final prespecified analysis was 42.1 months in the abiraterone and olaparib arm versus 34.7 months in the control arm (HR 0.81; 95% CI 0.67–1.00; *p* = 0.054) [33]. This was not significantly different between the two groups. In a subgroup analysis of OS in *BRCA*m patients, there was a trend towards benefit (median OS NR versus 23.0 months, HR 0.29; 95% CI 0.14–0.56) [34]. The combination of olaparib and abiraterone is FDA-approved for use in patients with *BRCA*-mutated mCRPC.

In the TALAPRO-2 phase 3 trial, patients with asymptomatic or mildly symptomatic mCRPC in the first-line treatment setting were randomised between talazoparib and enzalutamide versus placebo and enzalutamide [35]. The primary endpoint was radiologic PFS (rPFS) by BICR in the ITT population. At the time of primary analysis, the median rPFS in the ITT population was NR in the talazoparib and enzalutamide group versus 21.9 months for the control group (HR 0.63; 95% CI 0.51–0.78; *p* < 0.0001). In the HRRm population, the rPFS was superior with talazoparib and enzalutamide (27.9 months versus 16.4 months, HR 0.46; 95% CI 0.30–0.70; *p* = 0.0003). In the *BRCA*m subgroup, the HR for rPFS was 0.23; 95% CI 0.10–0.53; *p* = 0.0002. The OS data are still immature and awaited. The combination of talazoparib and enzalutamide is FDA-approved for use in HRRm mCRPC.

The MAGNITUDE study was a phase 3 study randomising patients with mCRPC to niraparib with abiraterone acetate and prednisone versus abiraterone acetate and prednisone with placebo [36]. Both patients who were HRRm and those who were HRR gene mutation negative were eligible. A prespecified futility analysis showed no benefit for niraparib and abiraterone acetate in the HRRm negative cohort, and further enrolment was stopped. The primary endpoint in the HRRm cohort was rPFS assessed by BICR first in the *BRCA1/2* group followed by HRRm patients. In the *BRCA*m population, the rPFS by BICR was 19.5 months versus 10.9 months, HR 0.55; 95% CI 0.39–0.78; *p* = 0.0007 demonstrating the superiority of niraparib and abiraterone. In the HRRm population, the rPFS by BICR was 16.7 months versus 13.7 months, HR 0.76; 95% CI 0.60–0.97; *p* = 0.0280. OS was not significantly improved with niraparib and abiraterone acetate and prednisone in both the *BRCA1/2* population and HRRm population. The combination of abiraterone acetate and prednisone with niraparib is FDA-approved for patients with *BRCA*m mCRPC.

In summary, it is important for all patients with mCRPC to undergo somatic and germline HRR gene testing to guide treatment decisions. Multiple options for PARPi monotherapy and combinations have shown PFS benefit in mCRPC. *BRCA*m remains the strongest predictor for the benefit of PARPi or PARPi combinations in mCRPC. HRRm is also a predictor for the benefit of PARPis in mCRPC; however, the PFS benefits are less marked and more inconsistent with non-*BRCA* HRRm compared to *BRCA*m. Given the lack of OS benefit with PARPi combinations and the effectiveness of single-agent PARPis as an alternative, considering cost and drug approvals when deciding on sequencing approaches is important. The results of some of the key PARPi trials in mCRPC are summarised below in Table 3.

### 2.5. Pancreatic Cancer

Pancreatic cancer is a deadly disease with surgery providing the only chance for cure. Unfortunately, only 15–20% of patients have resectable disease at diagnosis [37]. The remaining patients have either locally advanced or metastatic pancreatic cancer. Metastatic pancreatic cancer carries a poor prognosis with a median OS of less than 1 year even with intensive triplet-agent chemotherapy regimens [38,39]. The development of therapeutic resistance is common in pancreatic cancer due to its immunosuppressive tumour microenvironment (TME) [40]. Understanding the interaction between tumour cells and the TME is crucial to understanding the disease. The TME in pancreatic cancer comprises cancer-associated fibroblasts, tumour-associated macrophages and tumour endothelial cells that can promote tumour progression and metastases in various ways [41]. Gu et al. summarised ways in which patient-derived models and single-cell RNA sequencing could be used to study pancreatic tumour cells, their interaction with the TME, drug efficacy and the mutational heterogeneity within tumour cells and the TME [40]. These advanced techniques to study pancreatic cancer may help to develop our understanding of the disease, test drug efficacy and develop more effective therapeutics.

One of the advancements in treating metastatic pancreatic cancer has been the successful use of PARPis for g*BRCA*m patients. In the phase 3 POLO study, patients with metastatic pancreatic cancer with no progression of disease after at least 16 weeks of platinum-based chemotherapy who also were gBRCAm were randomised between olaparib maintenance versus placebo [42]. The study demonstrated a statistically significant benefit with olaparib for the primary endpoint of PFS (median PFS 7.4 months versus 3.8 months, HR 0.53; 95% CI 0.35–0.82; *p* = 0.004). At the final analysis of OS, there was no statistically significant benefit observed for olaparib over placebo (median OS 19.0 months versus 19.2 months, HR 0.83; 95% CI 0.56–1.22; *p* = 0.3487) [43]. However, there was Kaplan–Meier OS curve separation starting at 24 months with the estimated 3-year OS favouring olaparib over placebo numerically (33.9% versus 17.8%).

Rucaparib is another PARPi that has demonstrated efficacy in metastatic *BRCA*m or *PALB2* mutated pancreatic cancer. In a phase 2 single-arm trial, 46 patients with somatic or germline PV/LPV in *BRCA* or *PALB2* who did not have progression after 16 weeks of platinum-based chemotherapy were treated with rucaparib maintenance until disease progression or unacceptable toxicity [44]. Of these 46 patients, 42 were evaluable for mutations and consisted of 27 germline *BRCA2*, 7 germline *BRCA1*, 6 germline *PALB2* and 2 somatic *BRCA2* patients. The median PFS was 13.1 months and the median OS was 23.5 months. The ORR of patients with measurable disease was 41.7%. The ORR was similar in germline *BRCA2* (41%, 11 out of 27), germline *PALB2* (50%, 3 out of 6) and somatic *BRCA2* (50%, 1 out of 2). Although limited by small numbers, these comparisons suggest that metastatic pancreatic cancer patients with *PALB2* mutation and somatic mutations in *BRCA* also benefit similarly from PARPis.

In patients with metastatic pancreatic cancer and g*BRCA*m, olaparib maintenance should be discussed if there is no progression after 16 weeks of platinum-based chemotherapy given its PFS benefit.

## 3. Discussion

### 3.1. PARP Inhibitor Resistance

Despite the use of PARPis in patients with *BRCA1/2* PVs/LPVs or underlying HRD, some patients inadvertently develop PARPi resistance. This occurs through cancer cells developing various mechanisms to evade the effects of PARPis, rendering the treatment ineffective. The pathophysiological processes resulting in PARPi resistance can be broadly classified as homologous recombination-dependent or independent [45]. Homologous recombination-dependent mechanisms include reversion mutations, secondary DNA repair mutations, restoration of the HRR pathway and suppression of non-homologous end joining (NHEJ). Homologous recombination-independent mechanisms include alterations in the PARP1 protein, replication fork protection and the overexpression of PARPi drug efflux pumps [46,47].

Reversion mutations can occur via secondary intragenic mutations that restore HRR. Single and multiple mutations can occur, which restore the frameshift caused by the *BRCA1/2* PVs/LPVs. This leads to restoration of the open reading frame, allowing the synthesis of a functional BRCA1/2 protein [48,49,50,51]. Sakai et al. looked at secondary intragenic mutations in *BRCA2*-mutated cancer cell lines that restored the WT *BRCA2* reading frame. They demonstrated that all clones with secondary mutations were resistant to both cisplatin and PARPis [49]. In a retrospective analysis by Tobalina et al., 269 cases of reversion mutations in 86 patients with *BRCA1/2* mutations were identified. The majority of these secondary mutations came from patients with ovarian cancer treated with olaparib. These reversion mutations resulted in the correction of the original tumour mutation or re-established the *BRCA* open reading frame that was initially disrupted. Thorough analyses of the reversion mutations highlight that most amino acid sequences encoded by exon 11 in *BRCA1/2* were dispensable to *BRCA* function and thus conferred resistance to platinum-based chemotherapy or PARPis. Reversion mutations in other domains of *BRCA1* had less flexibility with relatively small deletions in the amino acid sequences resulting in the loss of function of the gene. Deletions were found to be the main mechanism of reversion mutations. Signature analysis of these deletions suggested that NHEJ-driven repair could be a major mechanism in generating reversions [52]. This could have therapeutic implications for the efficacy of compounds inhibiting DNA-PK, which is the key protein kinase involved in NHEJ [53,54].

The overexpression of *BRCA* can also occur through copy number gain or upregulation of the remaining functional allele in tumours with the somatic loss of *BRCA*. This was demonstrated in three patients with HGSOC with somatic *BRCA*m who had exceptional responses to olaparib lasting more than 5 years. The comparison of baseline exome, low-pass genome and RNA sequence analysis of tumours at diagnosis and upon relapse showed that in two of the patients, there was *BRCA* recovery through copy number gain and/or upregulation of the remaining functional allele [55].

Alternative DNA repair pathways such as the error-prone NHEJ pathway may be activated to compensate for the loss of the *BRCA*-dependent HRR and contribute to sensitivity to PARPis. The reactivation of HRR and resistance to PARPis can occur when NHEJ regulatory factors are disrupted, leading to the restoration of HRR [56,57]. In *BRCA1*-null mouse embryonic stem cells, the loss of TP53-binding protein 1 (53BP1) rescued proliferation defects in *BRCA1*-null cells, partially restored HRR in these cells and resulted in the loss of their cisplatin sensitivity [56]. The balance between HRR and NHEJ pathways is also maintained by miR-622, which limits NHEJ and promotes HRR [58]. Studies on a *BRCA1*-mutant cell line UWB1.289 showed that miR-622 levels inversely correlated with Ku70 and Ku80 transcripts. The Ku complex is essential for classical-NHEJ, and reduced expression results in the activation and rescue of the normally deficient HRR pathway of *BRCA1* mutated cells. The overexpression of miR-622 in *BRCA1*-mutant breast and ovarian cancer cell lines MDA-MB436 and UWB1.289 induced resistance to the PARPis olaparib and veliparib as well as to platinum chemotherapy [58].

Epigenetic modifications such as promoter methylation in *BRCA1* confer sensitivity to PARPis due to the silencing of *BRCA1* causing HRD [59,60,61]. The loss of promoter methylation in *BRCA1*, which can occur through active demethylation or the positive selection of less methylated tumour cells, may also contribute to PARPi resistance by recovering *BRCA*1 function and reactivating HRR [50,60,62,63]. Cancer cells may also acquire additional mutations in other DNA repair genes in the HRR pathway, such as *PALB2*, which may compensate for the loss of *BRCA* function. Secondary mutations in *RAD51C* and *RAD51D* can also restore HRR function and lead to PARP resistance [64].

PARP1 is a protein that is the primary target of PARPis and is responsible for 80–90% of poly ADP-ribosylation (PARylation) [65]. PARPis inhibit PARylation and trap PARP1 on DNA, resulting in replication fork collapse and eventually cell death [66]. Mutations in PARP1 can diminish the binding of PARPis. This allows PARP1 to maintain its functions and also diminishes PARP trapping, leading to PARPi resistance [46]. Pettitt et al. used a genome-wide CRISPR-Cas9 mutagenesis screen in mouse embryonic stem cell mutants resistant to talazoparib. Of the 24 resistant clones, 9 clones showed single-guided RNAs targeting PARP1 and absent PARP1 protein, demonstrating the importance of PARP1 in PARPi resistance [67]. Post-translational modifications of PARP1 such as phosphorylation by MET can also result in reduced binding by PARPis, resulting in resistance [68]. Du et al. showed that in TNBC cell lines, the use of c-MET inhibitors crizotinib or foretinib enhanced the sensitivity of the cell lines to the PARP inhibitors olaparib, rucaparib and veliparib [68]. Poly(ADP-ribose) glycohydrolase (PARG) degrades nuclear PAR and counteracts the effects of PARP1 [69]. By studying both PARPi-sensitive and PARPi-resistant *BRCA2* mutated mouse mammary tumours, Gogola et al. identified the loss of PARG as a major resistance mechanism. PARG depletion is associated with PARPi resistance in HRD tumours by restoring PARP1 signalling [69].

Replication fork instability is a major mechanism through which PARPis exert their cytotoxicity. Consequently, replication fork protection can result in PARPi resistance. MRE11 is involved in DNA double-strand repair, but in the setting of *BRCA*m, it may also precipitate uncontrolled degradation and fork collapse. In a study on *BRCA1/2* deficient mammary cells, the depletion of chromatin remodelers SMARCAL1, ZRANB3 and HLTF led to the prevention of strand degradation by MRE11, resulting in replication fork protection and PARPi resistance [70]. Pax2 transactivation domain-interacting protein (PTIP) is required to recruit MRE11 nuclease for replication fork degradation. PTIP deficiency thus protects the DNA strands from extensive degradation, resulting in reduced levels of chromosomal aberrations in *BRCA1/2* deficient cells, genomic stability and PARPi resistance [71]. EZH2, a histone methyl-transferase, is involved in replication fork degradation through recruiting MUS81 to the stalled fork [72]. The loss of expression of EZH2 in a murine *BRCA2-*deficient breast tumour model was associated with acquired PARPi resistance through replication fork stabilization [72].

The upregulation of drug efflux pumps can lead to PARPi resistance as well. Alterations in *ABCB1*, the gene encoding the multi-drug efflux pump MDR1 (P-glycoprotein 1), result in the increased expression and upregulation of MDR1 [73]. Upregulation of the P-glyocoprotein transporter can lead to PARPi resistance through increased drug extracellular translocation [74]. In vivo studies by Durmus et al. demonstrated that using small molecule inhibitors zosuquidar and Ko143 to inhibit the transporters ABCB1 and ABCG2 resulted in higher plasma and brain levels of rucaparib in mice [74]. This may be relevant for improving the effectiveness of PARPi therapy, treating brain metastases or micrometastases and overcoming PARPi resistance.

Data are limited in comparing the frequency of various mechanisms of PARPi resistance between different cancer types. However, a comparison of the different *BRCA* reversion mutations that occurred in breast cancer, prostate cancer and high-grade serous ovarian cancer (HGSOC) suggests that the specific reversion mutations and their frequencies pre- and post-PARPi exposure vary amongst tumour type [75]. In a study looking at *BRCA2* reversion mutations in mCRPC, a large genomic database identified 24 g*BRCA*m patients out of 1534 mCRPC patients undergoing circulating tumour DNA (ctDNA) testing. Of these 24 patients, 5 of them received PARPis or platinum chemotherapy and 2 of them (40%) had *BRCA2* reversion mutations [76]. Another clinical trial examining the acquisition of *BRCA* reversion mutations showed that 8 out of 97 HGSOC patients (8.2%) with g*BRCA*m or somatic *BRCA*m had *BRCA* reversion mutations prior to rucaparib treatment. After treating with rucaparib, an additional 8 out of 78 (10.3%) post-progression patients had *BRCA* reversion mutations that were not present in pre-treatment testing [77]. The limitation of these data is the small numbers that make cross-tumour comparisons challenging.

Much research has been undertaken to understand these pathways and develop novel strategies to overcome PARPi resistance. For example, the detection of RAD51 via immunohistochemistry staining has the potential to be a novel predictive biomarker for PARPi resistance [78]. Research investigating the molecular mechanisms of PARPi resistance has explored the use of combination therapy to enhance the anti-tumour effects of PARPis. Li et al. found that the use of enzalutamide in CRPC, a nonsteroidal antiandrogen, inhibits the expression of several genes associated with HRR in CRPC cell lines. Thus, combining PARPis with enzalutamide could promote cell death in CRPC cells [79]. The concurrent inhibition of other targets such as VEGFR, WEE1 and mTOR pathways have also been studied as means of overcoming PARPi resistance [80]. The combination of olaparib and bevacizumab has been proven efficacious in the PAOLA-1 trial [17]. WEE1 regulates the cell cycle in order to facilitate DNA repair. WEE1 inhibitors cause cells to enter the S phase with unrepaired DNA and may help to overcome PARPi resistance when used in combination [80]. The WEE1 inhibitor adavosertib with or without olaparib was investigated in a phase 2 non-comparative study in patients with epithelial ovarian cancer with documented progression on PARPis. The study showed that adavosertib had an ORR of 23% and median PFS of 5.5 months alone and an ORR of 29% and PFS of 6.8 months when combined with olaparib [81]. One of the limitations of this combination was the higher incidence of grade 3–4 toxicities, primarily haematological and gastrointestinal [81]. In vitro and in vivo studies of mTOR inhibitors combined with PARPis in BRCA-proficient TNBC have shown that mTOR inhibitors significantly suppressed HRR [82]. Combining everolimus and talazoparib in TNBC xenografts demonstrated enhanced efficacy compared to either treatment alone [82]. The concurrent administration of P-glycoprotein inhibitors with PARPis has been shown to increase the intracellular concentration of PARPis in colon cancer cells [83]. In a study on mice models, combining PARPis with a P-glycoprotein inhibitor following recurrence after PARPi treatment successfully re-sensitized some of these tumours to PARPis [84].

While combination treatment offers a potential strategy to overcome PARPi resistance, an improved understanding of the exact underlying mechanisms of resistance and how combination therapy overcomes them is required. More work and translational studies are required to identify predictive biomarkers and facilitate patient stratification for these novel therapeutics. While preclinical studies have shown many promising strategies to overcome PARPi resistance, another major challenge remains in demonstrating safety in human trials and subsequently confirming efficacy in phase 2 and 3 trials. This process is long, challenging and costly, and many therapeutic agents do not make it to clinical use in patients. Toxicities are another potential issue with combinatorial strategies that affect clinical applicability and hence need careful consideration as well.

### 3.2. PARP Inhibitor Toxicities

PARP inhibitors are generally well tolerated as a treatment. The most common side effects of PARPis are usually low grade; however, there are some uncommon but potentially serious side effects. Common class effect toxicities with PARPis include fatigue, anaemia, nausea and vomiting. Nausea tends to occur early on soon after starting PARPis, is usually low grade and improves over time [85]. It can be managed with anti-emetics and administration with a light meal. If these measures are not successful, then dose interruption or dose reduction are other strategies that can be used to manage it. All-grade fatigue is common with PARPis with 64% of patients in SOLO1 experiencing any-grade fatigue compared to 42% on placebo [86,87]. However, grade 3 to 4 fatigue on olaparib was only 4% versus 2% with placebo.

Haematologic toxicity is common with PARPis with anemia being the most common toxicity. The incidence of grade 3 to 4 anemia in the frontline ovarian trials was 22% with olaparib, 31% with niraparib and 29% with rucaparib [11,14,16]. Thrombocytopenia is more common with niraparib compared to the other PARPis with an incidence of 29% versus 7% with rucaparib and 1% with olaparib in the frontline maintenance ovarian cancer trials. The incidence of thrombocytopenia may be related to the volume of distribution (Vd) and bone marrow exposure as niraparib has a Vd of 1074/L versus 420/L for rucaparib and 158/L for olaparib [88]. The incidence of grade 3 to 4 neutropenia is not uncommon being 15% with rucaparib, 13% with niraparib and 9% with olaparib in ATHENA-MONO, PRIMA and SOLO1, respectively. Neutropenic fever is rare, however, and growth factors are not required for neutropenia. Blood counts should be monitored closely after starting PARPis especially with niraparib for which weekly blood count monitoring is recommended for the first month. Patients being started on niraparib should be started on 200 mg/day if they have a body weight of less than 77 kg and/or a baseline platelet count of less than 150,000/μL. For grade 3 and above cytopenias, PARPis should be interrupted and patients who have not had a recovery of blood counts to grade 1 or less after 4 weeks of interruption should be referred to a haematologist for bone marrow studies and further workup.

There are some notable differences in toxicities between the PARP inhibitors because of variations in their polypharmacology and off-target effects [88,89,90,91]. Niraparib can cause significant thrombocytopenia and neutropenia and also can cause hypertension and uncommonly arrhythmias [92]. Niraparib-induced hypertension may be caused by the off-target inhibition of the kinase DYRK1A, which may increase levels of neurotransmitters in the dopaminergic system [93]. Weekly blood pressure monitoring is recommended in the first 2 months after starting niraparib. The incidence of any grade hypertension was 17% in PRIMA with a 6% incidence of grade 3 or higher hypertension [14]. Rucaparib is associated with a higher incidence of transaminitis compared to the other PARP inhibitors with a 43% incidence of any grade elevation in ALT/AST and a grade 3 to 4 incidence of 11% [16]. Talazoparib, similar to niraparib, also has higher incidences of grade 3 and above thrombocytopenia (15%) and neutropenia (21%), as seen in the EMBRACA trial [5].

Uncommon but serious toxicities of interest include MDS, AML and pneumonitis. In a meta-analysis that included 5693 patients treated with PARPis, the incidence of MDS/AML was 0.73% across all PARPis compared to 0.47% in controls [94]. However, it should be noted that in the SOLO2 trial final analysis, MDS/AML incidence was 8% in the olaparib arm (5% MDS and 3% AML) versus 4% in the placebo arm [21]. This contrasts with the low incidence in the SOLO1 study, where the MDS/AML incidence at final analysis was 1.5% vs. 0.8% with olaparib versus placebo [13]. This highlights the significance of the number of prior chemotherapy treatment lines as a risk factor influencing the incidence of treatment-related MDS/AML.

Pneumonitis risk is increased with PARPi use, although the overall incidence remains low. In a meta-analysis of 5771 patients treated with a PARPi or control, the incidence of all-grade pneumonitis was 0.79% with PARPis versus 0.24% in those treated with control, odds ratio 2.68; 95% CI 1.38–5.47; *p* = 0.007 [95]. The median time to event onset for pneumonitis associated with PARPis was 81 days with most cases occurring during the first 6 months of treatment [95].

### 3.3. Beyond BRCA Mutation

Women with HGSOC should all be offered germline testing and have approximately a 20% chance of being found to have a PV/LPV using this method. Unfortunately, this approach may miss those who have somatic mutations in their tumour. Although both somatic and germline testing can be valuable in the diagnosis and management of carcinomas, there are many hurdles when it comes to testing. These include cost and accessibility to test assays, sufficiency of tumour tissue biopsy samples, physician familiarity and preference, and national and healthcare system guidelines.

In a cost–benefit analysis of somatic and germline testing performed by Kwon et. al, based on the cost of PARPis and test assays, tumour testing was found to be more costly than germline testing. It did, however, successfully identify more *BRCA1/2* PV/LPV and patients who are eligible for PARPis. After a price adjustment of test assays and drugs using a simulation model, the overall analysis became more cost-effective [96]. Eoh et al. were able to identify an additional 10% of patients that potentially could be eligible for PARPis after the addition of somatic testing by NGS [97]. Under a different healthcare model, HRD testing was shown to be cost-effective compared to germline testing and a cost-saving strategy compared to the sequence of tests in Italy [98]. The probabilistic sensitivity analysis performed by Rogoni et al. showed that the HRD test is cost-effective compared to *BRCA* testing in 98.5% of model simulations considering willingness to pay.

Our Cancer Genetics Service over at the National Cancer Centre previously piloted a study evaluating the impact of subsidies on cancer genetic testing uptake in Singapore [99]. Using a decision model, we estimated total spending on cancer management from the government’s perspective and found that the provision of subsidies (considering the financial status of individuals) leads to a considerable increase in the uptake rate of genetic testing. From a health policy point of view, subsidising genetic testing may potentially reduce the total costs of cancer management. This highlights the need for improved subsidies or reimbursement to lower costs of testing assays, which can lead to improved accessibility to therapeutics for those eligible. Early detection and personalized treatment can potentially reduce the need for more expensive and invasive interventions later on, ultimately saving healthcare resources.

In an Italian national survey of breast centres associated with Senonetwork by Tinterri et al., multiple issues were identified in performing genetic testing [100]. Cost and reimbursement issues was one of the problems identified in 11.9% of centres. However, other issues included reporting times in 35.7% of centres, execution times for pre-test counselling in 13.8% and other issues in 8.3%. In this survey, it was noted that only 48.6% of breast centres performed genetic testing in patients with TNBC. This is in contrast to the NCCN recommendation that patients diagnosed at any age with TNBC should undergo germline genetic testing. Hence, in addition to cost subsidies and reimbursement, educating healthcare providers on genetic testing indications, organizing the identification process of eligible patients and standardizing screening protocols are also crucial to increasing the rate of genetic testing.

The “Angelina Jolie” effect increased awareness with regard to germline genetic testing and the risk reduction surgery that is recommended for *BRCA1/2* PVs/LPVs [101]. For most oncologists, referring patients for genetic counselling has moved beyond the intention of prognostication and risk assessment for tumour types and into the field of therapeutics that can improve disease outcomes. Although *BRCA1/2* is the most common genetic variant detected on germline testing, it is prudent to recognise non-*BRCA* HRR-related genes such as *PALB2*, *ATM* and *RAD51* can also contribute to HBOC and other carcinomas. Alterations in these genes can also support risk reduction, preventive strategies and therapeutic potentials. Also, *BRCA1/2* PV/LPVs are more common in certain ethnic populations, such as Ashkenazi Jews. However, other genetic mutations may be more prevalent in other ethnic groups. Expanding genetic testing beyond *BRCA1/2* can help identify at-risk individuals in diverse populations.

For germline PVs/LPVs that are associated with breast cancer, the treatment paradigm has started to shift into assessing whether these are high, moderate or low penetrance genes that may result in hereditary breast cancer. Not all individuals with *BRCA*1/2 PVs/LPVs will develop breast or ovarian cancer. Similarly, individuals without mutations in these genes may still be at increased risk due to mutations in other genes. Looking beyond *BRCA1/2* can help identify individuals who are at risk but not captured by traditional genetic testing. As discussed during the *BRCA* Montreal Conference 2023, such an approach allows for the management of patients based on risk group rather than focusing on specific gene variants. *PALB2* PV/LPV, in addition to *BRCA1/2*, is also considered a high penetrance breast cancer susceptibility gene with an absolute lifetime risk of 44–63% [102]. As one of the HRD genes that can contribute to HBOC, risk reduction surgery for breast and ovarian cancer may be an option in carriers in addition to imaging surveillance.

The detection of germline PVs/LPVs beyond *BRCA* may also have treatment implications. The Talazoparib Beyond *BRCA* phase 2 study demonstrated the efficacy of Talazoparib, a PARPi, in advanced breast cancer with germline *PALB2* PV/LPV, showing activity within the context of HRD beyond *BRCA1/2* [103]. The TBCRC 048 phase 2 study recruited patients with metastatic breast cancer. Cohort 1 had germline mutations in non-*BRCA1/2* HRR genes, while cohort 2 had somatic mutations in non-*BRCA* HRR genes or somatic *BRCA1/2* mutation. Patients received olaparib until progression with a primary endpoint of ORR and a secondary endpoint of PFS. Of the 54 patients enrolled, 87% had mutations in *PALB2*, somatic *BRCA1/2*, *ATM* or *CHEK2*. In cohort 1, ORR was 33% and in cohort 2, ORR was 31%. Confirmed responses were seen only with germline *PALB2* (ORR 82%, median PFS 13.3 months) and somatic *BRCA1/2* mutations (ORR 50%, median PFS 6.3 months). There were no responses observed with *ATM* and *CHEK2* mutations [104]. In an expansion cohort of TBCRC 048, 24 metastatic breast cancer patients with germline *PALB2* mutation and 30 patients with somatic *BRCA*m were enrolled and received olaparib until progression. This expansion cohort confirmed the findings of the initial cohorts with an ORR of 75% and median PFS 9.6 months in the germline *PALB2* mutation group. In the somatic *BRCA*m cohort, the ORR was 36.7% and the median PFS was 5.6 months [105].

Similarly, for pancreatic cancer, retrospective studies have demonstrated response in up to 58% in *PALB2* PV/LPV patients with advanced disease treated with platinum-based chemotherapy [106]. Maintenance rucaparib has proven to be a safe and effective maintenance option for *PALB2* PV/LPV patients, in addition to *BRCA1/2*, with platinum-sensitive advanced pancreatic cancer in a phase 2 trial, and it is a current FDA approved-indication [44]. As described in the pancreatic cancer section, in this phase 2 trial, the ORR for rucaparib in germline *BRCA2* (41%) was similar to that in germline *PALB2* (50%) and somatic *BRCA2* (50%), suggesting a similar benefit with *PALB2* mutations, although the limitation was that numbers were low [44].

For prostate cancer, the *ATM* gene which is involved in DNA repair has been associated with an increased risk of developing prostate cancer. In the PROFOUND study, olaparib was shown to be effective in treating patients with mCRPC who had germline or somatic mutations in genes involved in DNA repair, including *ATM* [30]. Cohort A of the study which included *BRCA*m and *ATM* mutations was positive for PFS benefit compared to the physician’s choice of treatment with an HR of 0.34; 95% CI 0.25–0.47; *p* < 0.001. It should be noted, however, that responses were not the same with *BRCA*m and *ATM* mutation. While not a preplanned analysis that the study was powered for, subgroup analyses showed that the PFS HR was 0.41 (95% CI 0.13–1.39) for *BRCA1*, HR was 0.21 (95% CI 0.13–0.32) for *BRCA2* and HR was 1.04 (95% CI 0.61–1.87) for *ATM* mutation. For the HRRm genes in cohort B, PFS HRs were similarly less impressive: *CDK12* HR 0.74 (95% CI 0.44–1.31), *CHEK2* HR 0.87 (0.23–4.13), *PPP2R2A* HR 6.61 (95% CI 1.41–46.41) and *RAD54L* HR 0.33 (0.05–2.54) [29]. Another gene involved in the HRD pathway, *PALB2,* interacts with *BRCA2* to facilitate the repair of DNA double-strand breaks. Mutations in *PALB2* can impair this repair process, leading to genomic instability and an increased risk of cancer development including that of prostate cancer. Rucaparib, another PARPi, has also shown promise in treating prostate cancer patients with *PALB2* somatic mutations with a response rate of 100% in mCRPC as seen in the phase 2 TRITON2 study [107]. In this same study, no patients in the *ATM*, *CDK12* or *CHEK2* subgroups had an objective response by independent radiology review [107]. These results highlight differences in the effectiveness of PARPis in different HRR gene PVs/LPVs, although more prospective studies designed to assess this are needed.

In the exploratory subgroup analysis of PAOLA-1 for ovarian cancer, non-*BRCA* HRR gene alterations detected on gene panel testing did not derive PFS benefit with maintenance olaparib and bevacizumab [108]. Genes of interest included *BLM*, *BRIP1*, *RAD51C*, *PALB2*, and *RAD51D*, which are either directly or indirectly involved in the HRR pathway, with a GIS score of >= 42 [18]. One postulation was that there may be HR mechanisms not picked up in the current HRD testing assays, such as *RAD51C* hypermethylation that have been reported to be associated with PARPi response [60]. Such epigenetic changes may be reversed with ongoing PARPi treatment, resulting in resistance with subsequent treatment due to the reversion of hypermethylation. Nonetheless, the small number of non-*BRCA* genes in PAOLA-1 analysis may limit the precise nature of analysing their sensitivity. Prospective research of HRR genes that include *BRIP1*, *RAD51C*, or *RAD51D* with corresponding raised GIS is needed to evaluate their response to PARPis. An ad hoc analysis of the NOVA trial studied the effectiveness of niraparib in g*BRCA* WT patients with HRR defects [109]. Of the 283 patients who were *BRCA* WT, 41 patients (14.5%) had a loss of function mutation in at least one non-*BRCA* HRR gene. There were a total of 16 non-*BRCA* HRR genes that had alterations of which the most common were *RAD51C* (nine patients) and *BRIP1* (seven patients) with the rest having less than five patients with alterations. The median PFS of niraparib versus placebo in these 41 patients was 6.2 months versus 3.8 months, HR 0.31; 95% CI 0.13–0.77. In 18 out of the 41 patients who had biallelic HRRm, the median PFS was 15.7 months versus 4.5 months, HR 0.22; 95% CI 0.05–0.89. In the remaining 23 out of 41 patients with monoallelic or unknown allelic status, the median PFS was 4.8 months versus 3.7 months, HR 0.91; 95% CI 0.35–2.34. In the 242 patients with *BRCA* WT and HRR gene WT tumours, the median PFS was 7.4 months versus 4.2 months, HR 0.49; 95% CI 0.35–0.70. These data, while being an ad hoc analysis, strongly suggest that patients with non-*BRCA* HRRm benefit from PARPis especially those with biallelic mutations. This magnitude of benefit with niraparib appears to be greater than that with HRR WT tumours.

### 3.4. Beyond Hereditary Breast and Ovarian Cancer

Other than the role of HRD genes for HBOC, there should be a consideration for other hereditary predisposition syndromes in patients with breast and/or ovarian cancer. Lynch syndrome, which is caused by DNA mismatch repair deficiency (dMMR), is another inherited condition that increases the risk of several types of cancer, including colorectal, endometrial, ovarian, gastric, pancreatic, and urinary tract cancers. Female carriers also have a higher lifetime risk of developing breast cancer compared to the general population depending on the specific gene mutation (*MLH1*, *PMS2* etc.). Risk reduction surgery is also an option for carriers. Compared to those with HRR gene alterations, the therapeutic implications differ when treating dMMR carcinomas. Patients with dMMR or microsatellite instability-high (MSI-H) cancers respond well to immunotherapy such as pembrolizumab. The utility of the revised Amsterdam criteria cannot be underestimated, as it plays an important role in identifying potential patients with Lynch syndrome even in the setting of breast and/or ovarian cancer.

Occasionally, g*BRCA*m can also occur concurrently with other germline mutations such as *TP53* [110]. In one study that screened 82 patients with breast cancer and cutaneous melanoma, six patients were found to have PVs. Two had g*BRCA1*, two had a germline *CDKN2A* germline mutation, one had a co-occurring germline *TP53* mutation and g*BRCA2* mutation, while the last patient had a germline *TP53* mutation only [110]. This highlights the importance of panel testing rather than single gene testing given the significant clinical implications of discovering PVs in other genes, for example, *TP53*. More recently, a study by Usui et al. showed that germline PVs in nine genes (*APC*, *ATM*, *BRCA1*, *BRCA2*, *CDH1*, *MLH1*, *MSH2*, *MSH6* and *PALB2*) were associated with the risk of gastric cancer. Furthermore, this study showed that there was an interaction between Helicobacter pylori infection and PVs in HRR genes increasing the risk of gastric cancer (relative excess risk due to interaction 16.01; 95% CI 2.22–29.81; *p* = 0.02). By 85 years of age, the cumulative risk of gastric cancer was 45.5% in people with Helicobacter pylori infection and HRR PV versus 14.4% in those without this interaction [111]. These newer data highlight both the importance of panel testing to detect non-*BRCA* HRRm as well as the need for our screening practices of patients with *BRCA* and non-*BRCA* HRRm to evolve with new emerging data.

The role of PARPis is also being explored in patients without underlying germline *BRCA1/2* PV. In patients with endometrial cancer, the status of the mismatch repair (MMR) is often evaluated to assess the suitability for use of immunotherapy and to screen for underlying Lynch syndrome. The PFS benefit was demonstrated in the recent DUO-E study that evaluated the first-line use of durvalumab immunotherapy combined with platinum-based chemotherapy in patients with recurrent or advanced endometrial carcinoma [112]. The benefit was seen across patients regardless of MMR status, although the benefit of immunotherapy was greatest in the dMMR group. The study also evaluated the addition of PARPis with immunotherapy. In its predefined exploratory analysis, DUO-E also reported that the addition of olaparib to durvalumab enhanced PFS benefit in the proficient MMR (pMMR) subgroup.

The RUBY part 2 study studied the combination of niraparib and dostarlimab as maintenance following chemotherapy in advanced endometrial cancer as compared to placebo maintenance after chemotherapy. This study also met its primary endpoint, showing PFS benefit in both the overall and pMMR subgroups [113]. In contrast to the RUBY part 1 study where most of the overall population benefits of adding immunotherapy to chemotherapy were driven by the dMMR subgroup [114], in the RUBY part 2 study, the addition of PARPis and immunotherapy had similar benefits in the overall population and pMMR subgroup [113]. These studies reinforce that PARPis may be considered beyond patients with HRD/HRRm due to the possibility of synergism with combination therapy.

Several mechanisms have been described through which PARPis and immunotherapy may have synergistic effects. Preclinical models have shown that PARPi-based therapy synergizes with anti-programmed cell death protein 1 (PD-1) immunotherapy against both MSI-H and microsatellite stable (MSS) colon cancer models with a potential sensitizing effect of anti-PD-1 therapy against MSS tumours [115]. In an advanced serous ovarian cancer mouse model, olaparib use increased programmed death-ligand 1 (PD-L1) expression, which results in immunosuppressive effects in the TME [116,117]. Combining olaparib with an anti-PD-L1 immunotherapy exhibited notable effects in this model [116]. Preclinical studies have also shown that PARP inhibition causes DNA damage that results in increased neoantigen formation, higher tumour mutational burden and elevated immunogenicity [118]. PARPis use also activates the cGAS-STING innate immunity pathway, which results in various immunogenic effects and immune responses [119,120,121]. One of the resultant effects is an increased expression of the chemokines CCL5 and CXCL10, resulting in T-cell recruitment and an enhancement of lymphocyte function in the tumour [122,123,124]. This has important implications for the potential settings and tumours for which PARPis can be used. The use of combination therapy may also assist in overcoming the PARPi resistance that may develop within cancer cells.

### 3.5. Ongoing Trials in BRCA and Beyond BRCA

Much research is ongoing in the treatment of *BRCA*m cancers as well as cancers with alterations in other DNA repair genes. A few of these ongoing trials examining the use of PARPis in patients with *BRCA*m or HRRm cancer are summarised below in Table 4.

### 3.6. Future Directions with Technological Advances

With technological advances occurring rapidly in the field of science, some of these developments may provide potential applications in the diagnosis or treatment of *BRCA*-related cancers. Nanozymes combine the physiochemical properties of nanomaterials with the catalytic activity of natural enzymes. In comparison to natural enzymes, nanozymes have excellent catalytic efficiency and stability under extreme conditions. They may provide a cheap alternative to natural enzymes in multiple medical applications including point-of-care tests, biomarker detection and antibody-based tests [131]. Wang et al. designed a Pt@mesoporous silica core–shell nanoparticle whose peroxidase-like ability was blocked after DNA adsorption. Using this system, a single base mutation associated with the *BRCA1* gene was successfully detected [132]. The development of newer cheaper detection methods may help to improve the efficiency and reduce the cost of genetic testing in the future. The role of nanozymes in cancer therapy is also a developing area where several potential uses have been described. These include catalytic therapy through the generation of reactive oxygen species to damage tumour cells, starvation therapy, depletion of tumour oxygen supply and activation of therapeutics [133].

Vergara et al. described the use of clustered regularly interspaced short palindromic repeats/associated proteins (CRISPRs) and non-CRISPR-based gene drives that are used in population replacement and the population suppression of mosquitoes [134]. Using gene therapy to correct mutated tumour suppression genes such as *TP53* or *BRCA1* thus leading to cancer control has been attempted [135]. A phase 2 study examined using a vector of LXSN-BRCA1sv as therapy for ovarian cancer; however, the results were disappointing with no tumour responses, disease stabilization and poor vector stability [136]. This was accomplished prior to the era of CRISPR-based genome engineering however, and since then, huge advances in cellular therapy have been made. Hence, this may be another direction for development in the treatment of *BRCA*m cancers.

## 4. Conclusions

Identifying breast, ovarian, pancreatic and prostate cancer patients with *BRCA*m plays a crucial predictive role in selecting those who will benefit significantly from PARPi therapy. The use of PARPis in *BRCA*m HBOC-related cancers has resulted in significant PFS benefits. In high-risk HER2-negative g*BRCA*m early breast cancer, adjuvant olaparib improves iDFS and OS. In HER2-negative g*BRCA*m advanced breast cancer, olaparib and talazoparib were both shown to result in longer PFS and higher ORR compared to chemotherapy of the physician’s choice. In advanced HGEOC with *BRCA*m, frontline maintenance olaparib resulted in a significant survival benefit. Olaparib combined with bevacizumab, niraparib monotherapy and rucaparib monotherapy all demonstrated significant survival benefits when used as frontline maintenance in HRD-positive advanced HGEOC. In patients with recurrent platinum-sensitive HGEOC with *BRCA*m and no prior PARPis, olaparib, niraparib and rucaparib as maintenance therapies resulted in significant PFS benefits. In patients with mCRPC, *BRCA*m and one prior NHA, patients derived significant PFS benefit from the use of olaparib or rucaparib compared to the physician’s choice of treatment. Patients with mCRPC and HRRm have also benefitted from PARPi treatment strategies. Patients with metastatic pancreatic cancer with g*BRCA*m who are responding to first-line platinum-based chemotherapy derived PFS benefit from the use of olaparib.

Multiple PARPi resistance mechanisms can develop, and these can be classified into homologous recombination dependent or independent. Understanding the pathophysiology that contributes to such resistance may allow the development of novel therapeutics. Combination therapy appears to have promising results in emerging trials. Beyond *BRCA1/2*, performing tumour HRR gene testing in addition to germline testing increases initial costs but also increases the detection of gene mutations and can allow more patients to be eligible for and benefit from PARPis. While g*BRCA*m remains the strongest predictor of PARPi benefit, other predictive markers such as HRD in ovarian cancer and certain non-*BRCA* HRR gene alterations in breast, prostate and pancreatic cancer are also helpful in guiding therapeutic decisions and should be considered.

## Figures and Tables

**Table 1:** PARPis in advanced HER2-negative g*BRCA*m breast cancer.

Trial and Characteristics	OlympiAD	EMBRACA
Trial design	Phase 3 open-label	Phase 3 open-label
Randomization	2:1	2:1
Experimental arm	Olaparib	Talazoparib
Control	Chemotherapy of the physician’s choice (capecitabine, eribulin or vinorelbine)	Chemotherapy of the physician’s choice (capecitabine, eribulin, gemcitabine or vinorelbine)
Primary endpoint	PFS (BICR)	PFS (BICR)
Median PFS (Experimental vs. Control)	7.0 months vs. 4.2 months, HR 0.58; 95% CI 0.43–0.80; *p* < 0.001	8.6 months vs. 5.6 months, HR 0.54; 95% CI 0.41–0.71; *p* < 0.001
Median OS (Experimental vs. Control)	19.3 months vs. 17.1 months, HR 0.90; 95% CI 0.66–1.23; *p* = 0.513	19.3 months vs. 19.5 months, HR 0.85; 95% CI 0.67–1.07; *p* = 0.17
Overall response rate	59.9% vs. 28.8%	62.6% vs. 27.2%

**Table 2:** Frontline maintenance strategies including PARPis in advanced epithelial ovarian cancer.

Trial and Characteristics	SOLO1	PRIMA	ATHENA-MONO	PAOLA-1	DUO-O
*BRCA*m(%)	100%	30%	21%	30%	0%
HRD(%)	N/A	51%	43%	48%	39%
Experimental arm(s)	Olaparib maintenance	Niraparib maintenance	Rucaparib maintenance	Olaparib + Bevacizumab maintenance	Arm 2: placebo + durvalumab + bevacizumab maintenance Arm 3: olaparib + durvalumab + bevacizumab maintenance
Control	Placebo	Placebo	Placebo	Placebo + bevacizumab maintenance	Placebo + bevacizumab maintenance
Primary endpoint	PFS in ITT	PFS in HRD PFS in ITT	PFS in HRD PFS in ITT	PFS in ITT	PFS in HRD (arm 3 vs. arm 1) PFS in ITT (arm 3 vs. arm 1)
Median PFS (experimental vs. control) in ITT	56.0 months vs. 13.8 months, HR 0.33; 95% CI 0.25–0.43; *p* < 0.0001	13.8 months vs. 8.2 months, HR 0.62; 95% CI 0.50–0.76; *p* < 0.001	20.2 months vs. 9.2 months, HR 0.52; 95% CI 0.40–0.68; *p* < 0.0001	22.1 months vs. 16.6 months, HR 0.59; 95% CI 0.49–0.72; *p* < 0.001	24.2 months vs. 19.3 months, HR 0.63; 95% CI 0.52–0.76; *p* < 0.0001
Median PFS (experimental vs. control) in *BRCA*m	56.0 months vs. 13.8 months, HR 0.33; 95% CI 0.25–0.43; *p* < 0.0001	22.1 months vs. 10.9 months, HR 0.40; 95% CI 0.27–0.62	NR vs. 14.7 months, HR 0.40; 95% CI 0.21–0.75	37.2 months vs. 21.7 months, HR 0.31; 95% CI 0.20–0.47	N/A
Median PFS (Experimental vs. Control) in HRD	N/A	21.9 months vs. 10.4 months, HR 0.43; 95% CI 0.31–0.59; *p* < 0.001	28.7 months vs. 11.3 months, HR 0.47; 95% CI 0.31–0.72; *p* = 0.0004	37.2 months vs. 17.7 months, HR 0.43; 95% CI 0.28–0.66	37.3 months vs. 23 months, HR 0.49; 95% CI 0.34–0.69; *p* < 0.0001
Median OS (ITT)	NR vs. 75.2 months, HR 0.55; 95% CI 0.40–0.76; *p* = 0.0004 (*p* < 0.0001 required for statistical significance)	46.6 months vs. 48.8 months, HR 1.01; 95% CI 0.84–1.23; *p* = 0.88	Immature	56.5 months vs. 51.6 months, HR 0.92; 95% CI 0.76–1.12; *p* = 0.4118	Immature

**Table 3:** PARPi treatment strategies in mCRPC.

Trial and Characteristics	PROfound	TRITON3	PROpel	TALAPRO-2	MAGNITUDE
*BRCA*m and HRRm selection	Cohort A: *BRCA*m (57% of cohort) or ATM mutation Cohort B: HRRm	*BRCA*m (75%) ATM mutation (25%)	Not required 28% HRRm 11% *BRCA*m	Not required 21% HRRm 7% *BRCA*m	HRRm cohort 53% *BRCA*m
Prior treatment	At least 1 NHA Up to 1 prior taxane	At least 1 NHA No prior chemotherapy for mCRPC	Tx naïve in mCRPC setting No prior NHA in past 12 months Prior chemo in CSPC setting allowed	No prior mCRPC treatment Prior abiraterone or docetaxel in CSPC setting allowed	No prior mCRPC treatment except up to 4 months abiraterone Prior to taxane, enzalutamide, darolutamide or apalutamide in CSPC setting allowed
Experimental arm	Olaparib	Rucaparib	Olaparib + abiraterone	Niraparib + enzalutamide	Niraparib + abiraterone
Control	Physician’s choice of NHA (abiraterone or enzalutamide)	Physician’s choice of NHA (abiraterone or enzalutamide) or docetaxel	Placebo + abiraterone	Placebo + enzalutamide	Placebo + abiraterone
Primary endpoint	ibPFS in cohort A	ibPFS (ITT)	ibPFS (ITT)	rPFS (ITT)	rPFS in *BRCA*m rPFS in HRRm
Median ibPFS/rPFS (experimental vs. control) in overall population	5.8 months vs. 3.5 months, HR 0.49; 95% CI 0.38–0.63; *p* < 0.001 (cohort A + B)	10.2 vs. 6.4 months, HR 0.61; 95% CI 0.47–0.80; *p* < 0.001	24.8 vs. 16.6 months, HR 0.66; 95% CI 0.54–0.81; *p* < 0.001	NR vs. 21.9 months HR 0.63; 95% CI 0.51–0.78; *p* < 0.0001	16.7 vs. 13.7 months, HR 0.76; 95% CI 0.60–0.97; *p* = 0.0280
Median ibPFS/rPFS (experimental vs. control) in *BRCA*m	7.4 months vs. 3.6 months, HR 0.34; 95% CI 0.25–0.47; *p* < 0.001 (cohort A—included ATM)	11.2 vs. 6.4 months, HR 0.50, 95% CI 0.36–0.69	NR vs. 8 months, HR 0.24; 95% CI 0.12–0.45	HR for rPFS 0.23; 95% CI 0.10–0.53; *p* = 0.0002	19.5 vs. 10.9 months, HR 0.55; 95% CI 0.39–0.78; *p* = 0.0007
Median ibPFS/rPFS (experimental vs. control) in HRRm	5.8 months vs. 3.5 months, HR 0.49; 95% CI 0.38–0.63; *p* < 0.001 (cohort A + B)	N/A	NR vs. 13.9 months, HR 0.50; 95% CI 0.34–0.73	27.9 vs. 16.4 months, HR 0.46; 95% CI 0.30–0.70; *p* = 0.0003	16.7 vs. 13.7 months, HR 0.76; 95% CI 0.60–0.97; *p* = 0.0280
Median OS (experimental vs. control)	19.1 vs. 14.7 months, HR 0.69; 95% CI 0.50–0.97; *p* = 0.02 (cohort A) 17.3 vs. 14.0 months, HR 0.79; 95% CI 0.61–1.03 (cohort A + B)	24.3 months versus 20.8 months, HR 0.81; 95% CI 0.58–1.12; *p* = 0.21 (*BRCA*m) 23.6 months versus 20.9 months, HR 0.94; 95% CI 0.72–1.23 (ITT)	42.1 vs. 34.7 months, HR 0.81; 95% CI 0.67–1.00; *p* = 0.054 (ITT) NR vs. 23.0 months, HR 0.29; 95% CI 0.14–0.56 (*BRCA*m)	Immature	29.3 vs. 28.6 months, HR 0.88; 95% CI 0.58–1.34; *p* = 0.5505 (*BRCA*m) 29.3 vs. 32.2 months, HR 1.01; 95% CI 0.75–1.36; *p* = 0.9480 (HRRm)
FDA approval	mCRPC with HRR gene mutation and prior NHA	mCRPC with *BRCA*m and prior NHA and taxane chemotherapy	mCRPC with *BRCA*m	mCRPC with HRR gene mutation	mCRPC with *BRCA*m
European Commission approval	mCRPC with *BRCA*m and prior NHA	-	mCRPC in whom chemotherapy is not clinically indicated	mCRPC in whom chemotherapy is not clinically indicated	mCRPC with *BRCA*m

**Table 4:** Current recruiting and ongoing trials in *BRCA* and beyond *BRCA*.

Trial ID and Characteristics	NCT04584255 [125]	NCT06533384 [126]	NCT03025035 [127]	NCT03990896 [128]	NCT05340413 [129]	NCT04038502 [130]
Study title	Niraparib + Dostarlimab in *BRCA* Mutated Breast Cancer	PARPi or Capecitabine Combined with PD-1 Inhibitors as Adjuvant Therapy in High-Risk TNBC	Pembrolizumab in Combination with Olaparib in Advanced *BRCA*-Mutated or HDR-Defect Breast Cancer	Evaluation of Talazoparib, a PARP Inhibitor, in Patients With Somatic *BRCA* Mutant Metastatic Breast Cancer: Genotyping-Based Clinical Trial	Predicting Olaparib Sensitivity in Patients With Unresectable Locally Advanced/Metastatic HER2-Negative Breast Cancer. (RADIOLA)	Carboplatin or Olaparib for *BRcA*-Deficient Prostate Cancer (COBRA)
Study design	Phase 2	Phase 3	Phase 2	Phase 2	Phase 2	Phase 2
Cancer type	Breast cancer	Breast cancer	Breast cancer	Breast cancer	Breast cancer	Prostate cancer
Inclusion	Stage I–III HER2-negative breast cancer Measurable disease, planned for neoadjuvant treatment *gBRCA*m or germline *PALB2* mutation	High-risk TNBC patients with non-pCR after neoadjuvant chemo-immunotherapy	Advanced breast cancer, any receptor type g*BRCA*m or homology-directed repair (HDR) defect	Metastatic HER2-negative breast cancer Somatic *BRCA1/2* mutation Excludes those with g*BRCA*m	Advanced HER2-negative breast cancer Cohort 1: Germline or somatic *BRCA1*, *BRCA2*, *PALB2*, *RAD51C* or *RAD51D* deleterious mutation Cohort 2: Unknown or negative mutation status and a RAD51-foci low score in most recent biopsy	Metastatic CRPC Prior therapy with abiraterone acetate, enzalutamide, apalutamide or darolutamide Mutation in the homologous DNA repair pathway (*BARD1*, *BRCA1*, *BRCA2*, *BRIP1*, *CHEK1*, *FANCL*, *PALB2*, *RAD51B*, *RAD51C*, *RAD51D*, *RAD54L*)
Arms	Arm A: TNBC—niraparib + dostarlimab Arm B: TNBC—3-week lead in of niraparib monotherapy followed by combination therapy with dostarlimab Arm C: ER+/HER2-— Niraparib + Dostarlimab	Experimental arm: g*BRCA*m patients receive fuzuloparib + camrelizumab Patients without g*BRCA*m receive capecitabine + camrelizumab Control arm: camrelizumab monotherapy	Single arm: pembrolizumab + olaparib	Single arm: talazoparib	Both cohorts: olaparib	Arm 1: Carboplatin first-line followed by second-line olaparib on crossover Arm 2: Olaparib first-line followed by second-line carboplatin on crossover
Primary endpoint(s)	Tumour-infiltrating lymphocytes pCR rate	iDFS	ORR	Median PFS	ORR in both RAD51-foci low tumours and RAD51-foci high tumours	PFS-first-line (PFS-1L)
Estimated primary completion	17 July 2025	31 December 2026	8 October 2024	31 July 2025	April 2024	29 August 2025

## Data Availability

No new data were created or analysed in this study. Data sharing is not applicable to this article.

## Abbreviations

ADP	Adenosine diphosphate
AML	Acute myeloid leukaemia
ASCO	American Society for Clinical Oncology
BICR	Blinded independent central review
ctDNA	Circulating tumour DNA
CPS + EG	Clinical and pathological stage plus oestrogen receptor and nuclear grade
CRISPR	Clustered regularly interspaced short palindromic repeats/associated proteins
CR	Complete response
CI	Confidence interval
dMMR	DNA mismatch repair deficiency
FDA	Food and Drug Administration
g*BRCA*m	Germline *BRCA* mutation
GIS	Genomics instability score
HR	Hazard ratio
HBOC	Hereditary Breast and Ovarian Cancer
HGEOC	High-grade epithelial ovarian cancer
HGSOC	High-grade serous ovarian carcinoma
HRR	Homologous recombination repair
HRRm	Homologous recombination repair gene mutations
HRD	Homologous repair deficiency
ibPFS	Imaging-based progression-free survival
ITT	Intention to treat
iDFS	Invasive disease-free survival
LPV	Likely pathogenic variant
LOH	Loss of heterozygosity
MSI-H	Microsatellite instability high
mCRPC	Metastatic castrate-resistant prostate cancer
MDS	Myelodysplastic syndrome
NGS	Next-generation sequencing
NHEJ	Non-homologous end joining
NHAs	Novel hormonal agents
NR	Not reached
ORR	Overall response rate
OS	Overall survival
PR	Partial response
PV	Pathogenic variant
PTIP	Pax2 transactivation domain-interacting protein
PAR	Poly ADP-ribose
PARylation	Poly ADP-ribosylation
PARG	Poly ADP-ribose glycohydrolase
PARP	Poly adenosine diphosphate ribose polymerase
PARPi	Poly adenosine diphosphate ribose polymerase inhibitor
pMMR	Proficient DNA mismatch repair
PD-1	Programmed cell death protein 1
PD-L1	Programmed death-ligand 1
PFS	Progression-free survival
rPFS	Radiologic progression-free survival
TNBC	Triple-negative breast cancer
t*BRCA*m	Tumour *BRCA* mutation
TME	Tumour microenvironment
53BP1	TP53-binding protein 1
Vd	Volume of distribution
WT	Wild type
